# Adjust Neuronal Reactions to Pulses of High-Frequency Stimulation with Designed Inter-Pulse-Intervals in Rat Hippocampus In Vivo

**DOI:** 10.3390/brainsci11040509

**Published:** 2021-04-16

**Authors:** Lvpiao Zheng, Zhouyan Feng, Yifan Hu, Zhaoxiang Wang, Yue Yuan, Gangsheng Yang, Chuchu Lu

**Affiliations:** Key Laboratory of Biomedical Engineering of Education Ministry, College of Biomedical Engineering and Instrument Science, Zhejiang University, Hangzhou 310007, China; zhenglvpiao@zju.edu.cn (L.Z.); huuyifan@zju.edu.cn (Y.H.); wangzhaoxiang@zju.edu.cn (Z.W.); yuanyue99@zju.edu.cn (Y.Y.); yanggscn@zju.edu.cn (G.Y.); luchuchu@zju.edu.cn (C.L.)

**Keywords:** neuronal stimulation, varying inter-pulse intervals, population spikes, mapping model, design algorithm

## Abstract

Sequences of electrical pulses have been applied in the brain to treat certain disorders. In recent years, altering inter-pulse-interval (IPI) regularly or irregularly in real time has emerged as a promising way to modulate the stimulation effects. However, algorithms to design IPI sequences are lacking. This study proposed a novel strategy to design pulse sequences with varying IPI based on immediate neuronal reactions. Firstly, to establish the correlationship between the neuronal reactions with varying IPIs, high-frequency stimulations with varying IPI in the range of 5–10 ms were applied at the alveus of the hippocampal CA1 region of anesthetized rats in vivo. Antidromically-evoked population spikes (APS) following each IPI were recorded and used as a biomarker to evaluate neuronal reactions to each pulse. A linear mapping model was established to estimate the varied APS amplitudes by the two preceding IPIs. Secondly, the mapping model was used to derive an algorithm for designing an IPI sequence that would be applied for generating a desired neuronal reaction pre-defined by a particular APS distribution. Finally, examples of stimulations with different IPI sequences designed by the algorithm were verified by rat experiments. The results showed that the designed IPI sequences were able to reproduce the desired APS responses of different distributions in the hippocampal stimulations. The novel algorithm of IPI design provides a potential way to obtain various stimulation effects for brain stimulation therapies.

## 1. Introduction

The application of electrical pulse sequences in the brain has shown promise for treating certain brain disorders [1,2]. Various stimulation paradigms have been developed to generate diversified effects to meet the demands for extending the application [2,3]. One major strategy for the development of the stimulation paradigm is to program the parameters of pulse sequences [4,5].

Usually, electrical stimulations in brain, such as deep brain stimulation (DBS), utilize continuous sequences of high-frequency stimulation (HFS) of pulses around 100 Hz. Therapeutic efficacies may be adjusted by programming pulse parameters, such as intensity (i.e., the amplitude of voltage or current pulses), pulse width (common 60–150 μs), and pulse frequency (i.e., the repetition rate of pulses) [6,7]. Traditionally, once the parameters are set, the HFS is applied with a fixed pulse frequency, i.e., with a fixed inter-pulse-interval (IPI) during the entire stimulation duration. However, an emerging way to modulate the stimulation effects is to design temporal patterns of pulses, that is to vary IPI regularly or irregularly in real time [8,9]. For instance, studies have shown that stimulations with irregular IPI can suppress seizures more effectively than stimulations with regular IPI in the basolateral amygdala of rats [10]. HFS sequences with randomly varying IPI of Poisson distribution in rat hippocampus can be more efficient to reduce the number of spontaneous seizures compared to regular HFS [11]. In addition, stimulations with certain irregular patterns of IPI may ameliorate motor symptoms and suppress pathological rhythmic activity in the basal ganglia more effectively than stimulations of regular IPI [12]. Thus, HFS sequences with randomly varying IPI provide a promising way to generate different neuronal reactions to meet the demands for treating different diseases. However, there is a lack of approach for designing pulse sequences with varying IPI, but trial.

The patterns of pulse sequences with randomly varying IPI are infinite. Previous studies have exploited IPI sequences randomly varying with specific distributions (e.g., uniform, Poisson, and Gamma distributions) [10,11,12,13,14], or IPI sequences mimicking the firing pattern of neurons [15,16], as well as IPI sequences according to the results of modeling computations and behavior outcomes of stimulations in animal experiments and clinic trials [17,18]. However, to our knowledge, there have been no algorithms reported to design the varying IPI sequences based on the direct neuronal reaction to each stimulation pulse. 

Commonly used frequency range of pulses, including the mean frequency of varying IPI, is about ~100–200 Hz [3,5,11], corresponding to an IPI about 5–10 ms. Previous studies have shown that HFS with a frequency in such range may prevent the propagation of pathological neural signals from upstream and generate new neuronal activity that would spread to the downstream areas [19,20]. The neuronal reaction directly induced by stimuli is the origin of final outcomes of the stimulations. HFS with different patterns of varying IPI could induce different patterns of neuronal firing, resulting in different stimulation effects. Here we propose an approach to design patterns of IPI sequences based on direct neuronal reactions. 

A pulse can activate a population of neurons close to the stimulation electrode. We quantified the direct neuronal reactions by antidromically-evoked population spikes (APS) induced by axonal stimulations, because axon is the neuronal structure with the lowest threshold to respond pulse stimuli [21,22]. HFS induced firing in axons can propagate in two directions: downstream to the terminals and synapses, and upstream to the cell bodies to generate APS. Therefore, APS can be used to evaluate the direct neuronal reactions without involving synaptic transmissions [23].

In this work, for the objective of designing IPI sequences based on neuronal reactions, we firstly created a computational mapping model to describe the relationship between the varying IPI and the direct neuronal reactions to pulses. Secondly, we used the model to develop an algorithm for designing IPI sequences for target neuronal reactions. Data from rat experiments were used to create the model and to validate the algorithm. The amplitude of APS was used as a biomarker to evaluate the direct neuronal responses to the HFS pulses. The APS waveforms were recorded extracellularly in the cell-body layer of pyramidal neurons in rat hippocampal CA1 region in vivo during HFS with varying IPI applied on the axons of neurons. The study provides a novel strategy for designing patterns of pulse sequences to obtained diverse effects in brain stimulations.

## 2. Materials and Methods

### 2.1. Animal Surgery

**Figure 1:**
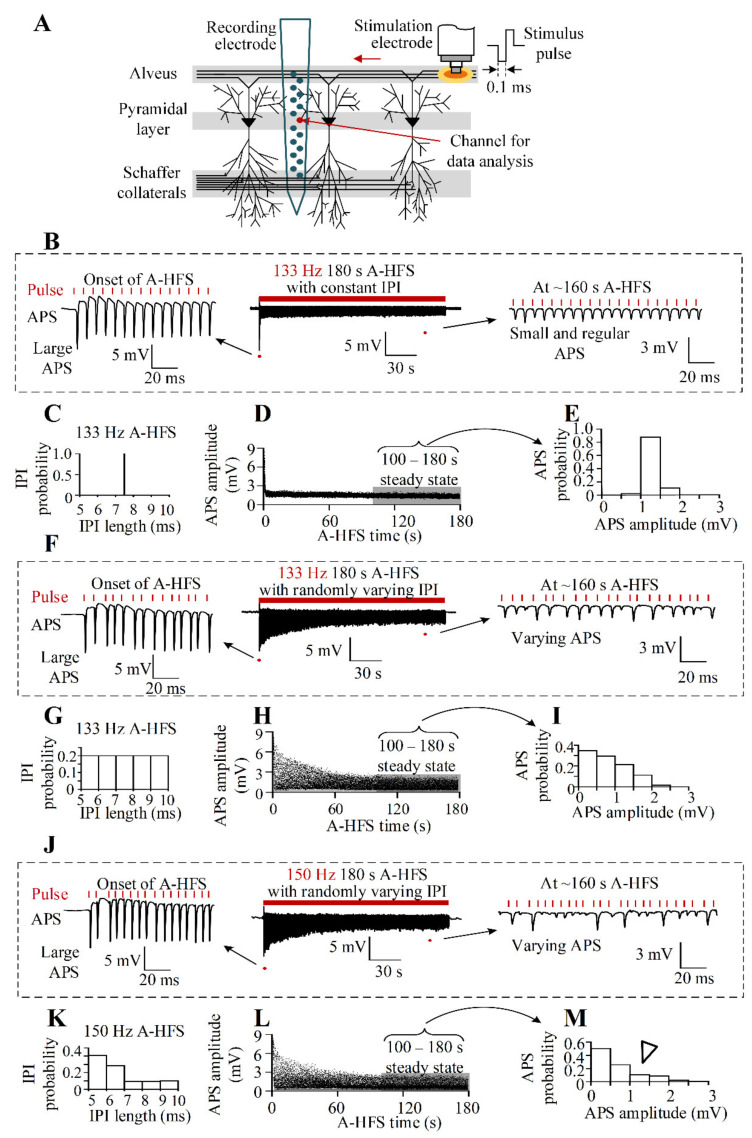
Different neuronal responses induced by 3-min A-HFS sequences with constant IPI 7.5 ms or with randomly varying IPI in a range of 5–10 ms but with different distributions. (**A**) Schematic diagram of the locations of recording electrode and antidromic stimulation electrode in the rat hippocampal CA1 region. (**B**–**M**) Examples of neuronal responses to three types of A-HFS sequences with a mean pulse frequency of 133 Hz (**B**–**E** and **F**–**I**) and 150 Hz (**J**–**M**), including the recordings of antidromically-evoked population spikes (APS) with the expanded APS waveforms at the onset and at the steady-state periods of A-HFS (**B**,**F,J**), the distribution of varying IPI (**C**,**G**,**K**), the amplitudes of each evoked APS during the whole A-HFS (**D**,**H,L**), and the distribution of APS amplitudes during the 100–180 s steady-state period of A-HFS (**E**,**I**,**M**). The hollow triangle in (M) denotes APSs with medium amplitudes.

The experiment protocol was approved by the Institutional Animal Care and Ethics Committee, Zhejiang University (Ethical approval code 14730 Zhejiang University, 1 March 2019). Thirty-seven male Sprague Dawley rats (250–350 g) were used under anesthetic with urethane (1.25 g/kg, i.p.). The details of the experimental procedures were reported previously [24]. In brief, after the rat was confined to a stereotaxic apparatus and part of the left skull was removed, a recording electrode array (#Poly2, NeuroNexus Technologies, Inc., Ann Arbor, MI, USA) was positioned in the hippocampal CA1 region (AP −3.5 mm; ML 2.7 mm; DV ~2.5 mm). To antidromically evoke population spikes in the CA1 region around the recording electrode, a bipolar concentric stimulation electrode (#CBCSG75, FHC, Inc., Bowdoin, ME, USA; diameters: inner pole 75 μm, outer pole 250 μm) was positioned at alveus (AP −4.8 mm; ML 2.7 mm; DV ~2.3 mm), the efferent fiber of the CA1 pyramidal neurons (Figure 1A). 

### 2.2. Stimulation

Stimuli of biphasic current pulses were generated by a programmable stimulator (Model 3800, A-M System, Inc., Sequim, Washington, DC, USA) with a width of 100 μs/phase and an intensity of 0.3 or 0.4 mA. The range of varying IPI was 5–10 ms with a temporal resolution of 0.05 ms. Pulse sequences with various distributions and orders of IPIs were created by a custom-made MATLAB program and were loaded into a custom-made LabVIEW program. The LabVIEW program controlled a USB-6251 DAQ card (National Instruments, Austin, TX, USA) to output a pulse sequence to trigger the 3800 stimulator to generate a required HFS sequence for antidromic stimulation. The duration of antidromic HFS (A-HFS) was 3 mins. The interval between successive A-HFS trains was longer than 20 mins to allow a complete recovery of neuronal state from previous A-HFS, which was confirmed by the recovery of evoked APS to baseline level.

The following groups of A-HFS sequences with varying IPI in the range of 5–10 ms were used in this study. Group 1 included eight different A-HFS sequences with a uniform distribution of randomly varying IPIs and with an identical mean pulse frequency of 133 Hz. They were applied in rats to acquire the experimental data of APS amplitudes for establishing the mapping model. Group 2 included A-HFS sequences of 133 and 150 Hz with randomly varying IPI, as well as 133 Hz A-HFS with gradually varying IPI. They were applied in rats to evaluate the predictions of the mapping model. Group 3 included two A-HFS sequences that were designed by an algorithm for obtaining two different types of desired neuronal reactions in rats (see the Results Section for the details of the algorithm).

### 2.3. Recording and Data Analysis

The raw electrical signals collected by the recording electrode were amplified 100 times by a 16-channel amplifier (Model 3600, A-M System, Inc., Sequim, Washington, DC, USA) with a band-pass range of 5–5000 Hz. The amplified signals were then sampled by a data acquisition system (Model PL3516, ADInstruments, Inc., Bella Vista, NSW, Australia) with a sampling rate of 20 kHz.

The stimulation artifacts in the recordings were removed by a custom-made MATLAB program [25]. The amplitude of APS evoked by each pulse of A-HFS was measured with a detection threshold of amplitude 0.1 mV and was then normalized by the amplitude of the first APS evoked at the onset of A-HFS. The IPIs preceding the current evoked APS (i.e., 1-back IPI, 2-back IPI, and k-back IPI) were termed IPI_1_, IPI_2_, and IPI_k_, respectively. The Pearson correlation coefficient (*R*) between the APS amplitudes of experimental data and the APS predicted by specific calculations of preceding IPIs was used to evaluate different mapping models between the current APS and its preceding IPIs. 

The root mean square error (RMSE) of APS amplitudes and the ratio of correct predictions of APS alterations (increase or decrease of amplitudes) were used as indexes to evaluate the predictions of mapping models or to evaluate the differences between realized values and pre-set values. Because the RMSE would be the standard deviation (SD) of the APS amplitudes when a mean APS amplitude was simply used to make the prediction, the mean RMSEs of the prediction model were compared with the mean SDs of the experimental APS amplitudes to show the prediction performance of the mapping model as well.

By using the mapping model, a design algorithm was derived to calculate the IPIs of an A-HFS sequence from a pre-set APS distribution, reversely. The detail of the algorithm is closely related with the results of modelling thereby being described in the Results Section.

One-way ANOVA with post hoc Bonferroni test or paired *t-test* were used to determine the statistical significances of differences among or between data groups. “*n*” represents the number of rats.

## 3. Results

### 3.1. Establish a Mapping Model for the Amplitudes of the Population Spikes Evoked by A-HFS Pulses

During a 133 Hz A-HFS with a constant IPI of 7.5 ms, large APS waveforms with similar amplitudes (~8.3 mV) were evoked by each pulse at the initial period of A-HFS (Figure 1B,C). However, after seconds of stimulation, the amplitudes of evoked APSs decreased rapidly and then were stable till the end of A-HFS. During the steady period of late A-HFS 100–180 s (Figure 1D), most APS amplitudes were about 1.5 mV (Figure 1E).

During an A-HFS (mean 133 Hz) with randomly varying IPI of a uniform distribution, large APS waveforms with similar amplitudes were also evoked by each pulse at the initial period of A-HFS despite the variations in IPI (Figure 1F,G). However, after seconds of stimulation, the amplitudes of evoked APSs varied substantially with the varying IPIs till the end of A-HFS. During the steady period of late A-HFS 100–180 s (Figure 1H), the distribution probability of APS amplitudes decreased with the increase of APS amplitudes approximately linearly (Figure 1I). 

During another A-HFS with varying IPI of a different distribution in the identical range of 5–10 ms but a mean pulse frequency of 150 Hz, the initial large APSs remained. However, the distribution of APS amplitudes in the steady period changed accordingly (Figure 1J–M). The distribution probability of APS amplitudes decreased more rapidly with the increase of APS amplitudes, resulting in a decrease of the amount of medium APSs (indicated by the hollow triangle in Figure 1M).

Because an APS potential waveform is formed by the integration of the synchronous discharges of a population of neurons, the change in the APS amplitudes can indicate a change in the number of discharging neurons activated by a pulse. The above results indicate that varying IPI patterns even within an identical time range may alter the firing pattern of neurons, which suggests a way to design pulse sequences for different neuronal reactions. 

Previous studies have shown that during the steady-state period of A-HFS, the amplitude of evoked APS correlated with the lengths of immediately preceding IPIs [13,26]. To design the required sequence of IPIs for a desired distribution of evoked APS, we first established a mapping model between the APS amplitude and its preceding IPIs.

**Figure 2:**
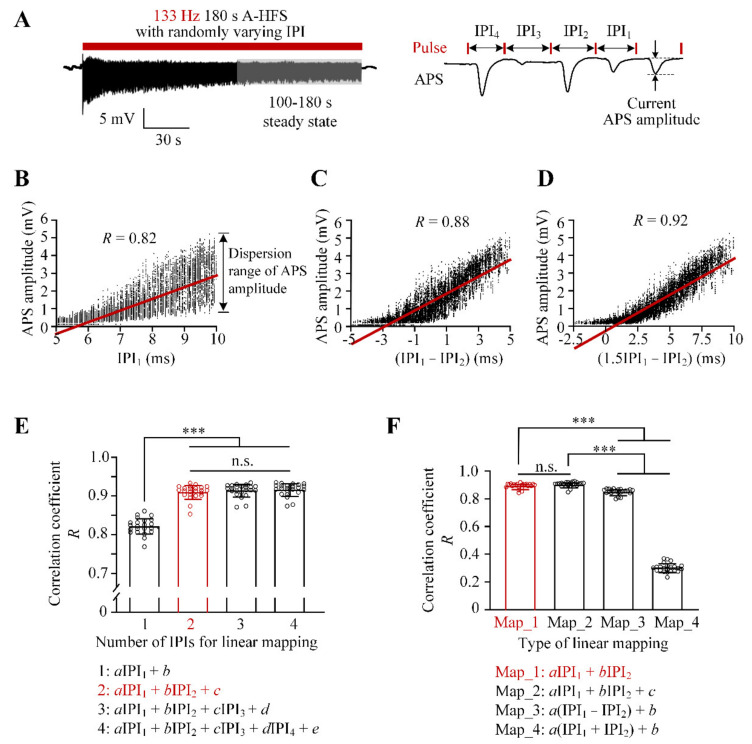
Comparison of different mapping models describing the relationship between the amplitudes of evoked APS and the preceding IPIs. (**A**) *Left*: evoked APS during an A-HFS sequence with uniformly varying IPI. *Right*: illustration of the current APS and its preceding IPIs (IPI_1_, IPI_2_, IPI_3_ and IPI_4_). (**B**–**D**) Examples of the correlations of APS amplitudes to IPI_1_, (IPI_1_ − IPI_2_) and (1.5IPI_1_ − IPI_2_) with their correlation coefficient (*R*), respectively. The APS amplitudes were collected from the 100–180 s steady-state period of A-HFS shown in (**A**). (**E**) Comparison of the *R* values for the linear mappings of APS amplitudes with different numbers of preceding IPIs. (**F**) Comparison of the *R* values for the different types of linear mappings with the two preceding IPIs (IPI_1_ and IPI_2_). *** *p* < 0.001; n.s., not significant; post hoc Bonferroni tests after ANOVA for the data from *n* = 23 rats.

Take one of the 133 Hz A-HFS with randomly varying IPI for example (Figure 2A), during the steady-state period, the APS amplitude increased with the increase of the preceding IPI_1_ (*R* = 0.82). However, the dispersion of APS amplitudes also increased with the increase of IPI_1_ (Figure 2B). The correlation coefficient increased to *R* = 0.88 for a mapping of APS amplitude with a calculation (IPI_1_ − IPI_2_) (Figure 2C), and further increased to *R* = 0.92 for a calculation (1.5IPI_1_ − IPI_2_) (Figure 2D) with a decrease of dispersions. 

To compare different mapping models of APS amplitudes, we made least square fittings for the experimental APS data by utilizing up to four preceding IPIs. The data were collected from 23 rats that received eight different sequences of 133 Hz A-HFS with a same uniform distribution of varying IPI as shown in Figure 1C. For the four types of mappings using one to four IPIs (Figure 2E), the mean *R* value significantly increased from *R* = 0.82 ± 0.02 for the mapping (*a*IPI_1_ + *b*) to *R* = 0.91 ± 0.02 for the mapping (*a*IPI_1_ + *b*IPI_2_ + *c*) (ANOVA *F*_3,88_ = 152, *p* < 0.001; post hoc Bonferroni test, *p* < 0.001, *n* = 23). Then the mean *R* values did not change significantly for the mappings with three or four preceding IPIs. Therefore, the two preceding IPIs (IPI_1_ and IPI_2_) were used to predict the APS amplitudes (Figure 2E, red). 

Next, four types of linear mappings with IPI_1_ and IPI_2_ were examined (Figure 2F). The mean *R* = 0.90 ± 0.02 for the mapping (*a*IPI_1_ + *b*IPI_2_) was similar to the mean *R* = 0.91 ± 0.02 for the mapping (*a*IPI_1_ + *b*IPI_2_ + *c*). The RMSE of the two mappings were similar as well (0.0349 ± 0.0075 vs. 0.0331 ± 0.0074). The *R* values of both mappings were significantly greater than the *R* values of the other two mappings [*a*(IPI_1_ − IPI_2_) + *b*] (*R* = 0.86 ± 0.02) and [*a*(IPI_1_ + IPI_2_) + *b*] (*R* = 0.31 ± 0.03) (ANOVA *F*_3,88_ = 3709, *p* < 0.001; post hoc Bonferroni tests, *p* < 0.001, *n* = 23). Therefore, the linear mapping (*a*IPI_1_ + *b*IPI_2_) was selected for conciseness (Figure 2F, red). 

Finally, the parameters *a* and *b* in the mapping model were determined by using least square fittings for the normalized APS amplitudes. The statistical values were *a* = 0.0409 ± 0.0092 and *b* = −0.0273 ± 0.0067 (*n =* 23), respectively. Therefore, we had the normalized APS amplitude (NAA):

NAA = 0.027(1.5IPI_1_ − IPI_2_) (1)

The value of the equation was set to 0 once an IPI_1_ and/or an IPI_2_ in the range of 5–10 ms resulted in a normalized APS amplitude smaller than 0. Thus, the final mapping model was:

NAA = max [0.027(1.5IPI_1_ − IPI_2_), 0] (2)

This mapping model was established based on the neuronal responses to the A-HFS with randomly varying IPIs distributed uniformly. Next, we evaluated the predictions of the mapping model for different distributions and different orders of IPIs varying in the identical range of 5–10 ms.

### 3.2. Predict Neuronal Responses to A-HFS with Varying IPI

**Figure 3:**
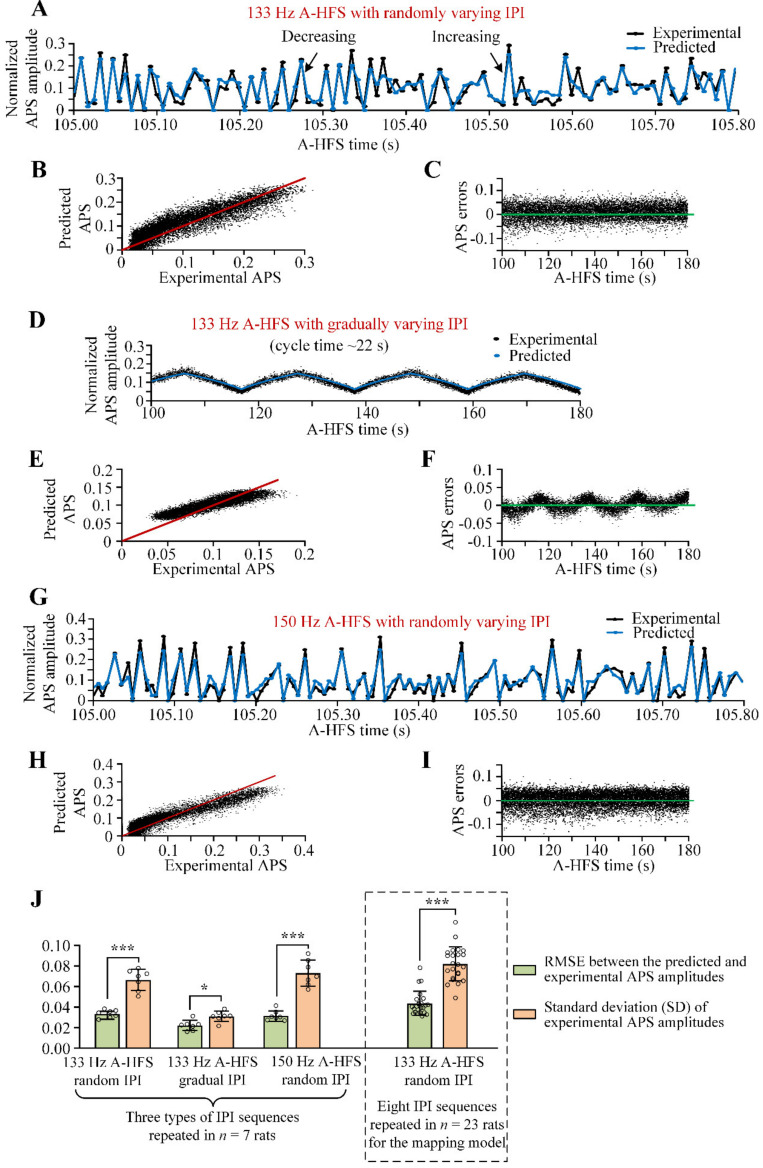
Using the mapping model of normalized APS amplitude (NAA) = max [0.027 × (1.5 × IPI_1_ − IPI_2_), 0] to predict the neuronal responses to A-HFS with different types of varying IPI. (**A**–**I**) Comparison of the predicted APS amplitudes and experimental APS amplitudes (normalized) during a 133 Hz A-HFS sequence with randomly varying IPI (**A**–**C**), during a 133 Hz A-HFS sequence with gradually varying IPI (**D**–**F**) and during a 150 Hz A-HFS sequence with randomly varying IPI (**G**–**I**). The predicted APS amplitudes followed the experimental APS amplitudes in an episode of A-HFS (**A**,**D**,**G**). The data points distribute around the diagonal line in the scatter plots of the predicted data (vertical coordinate) vs. the experimental data (horizontal coordinate) (**B**,**E**,**H**). The APS errors (predicted APS amplitude minus experimental APS amplitude) of each evoked APS are shown along the A-HFS time (**C**,**F**,**I**). (**J**) Comparisons between the RMSE of predicted APS and the SD of experimental APS (normalized) for the three different types of AHFS shown in (**A**–**I**), as well as for the other eight IPI sequences of 133 Hz A-HFS with randomly varying IPI for the mapping the model. * *p* < 0.05, *** *p* < 0.001, paired *t*-test between RMSE and SD, *n* = 7 or *n* = 23.

Three types of A-HFS sequences were used to verify the mapping model (Equation (2)). First, for a 133 Hz A-HFS sequence with randomly varying IPI of a uniform distribution, the mapping model was able to accurately predict the amplitudes of APS during A-HFS (Figure 3A–C). The A-HFS sequence was different from the sequences used for establishing the mapping model. The predicted data correctly followed 88.6 ± 1.1% (*n =* 7) of the APS alterations (increasing or decreasing) (Figure 3A). The consistency of the predicted APS to the experimental APS was shown clearly in the scatter plot (Figure 3B). Most (90.5%) of the errors of predicted APS amplitude (i.e., the predicted value minus the experimental value) were within a range of ±0.05 of the normalized APS amplitude (Figure 3C). 

Second, when the same set of IPI was arranged orderly to form an A-HFS of gradually varying IPI, the predicted data of the mapping model were also able to follow the experimental data accurately (Figure 3D). Most (99.9%) of the prediction errors were within a range of ±0.05 of the normalized APS amplitude (Figure 3E,F). For several successive pulses in a short period of A-HFS with the gradually varying IPI, the stimulation is equivalent to constant IPI [26]. Therefore, the result indicated that the model was also able to predict the APS generated by A-HFS of slowly varying IPI or constant IPI.

Third, when the A-HFS sequence was changed to a mean frequency of 150 Hz with IPI still randomly varying in the range 5–10 ms, the predicted APS correctly followed 88.8 ± 2.2% (*n =* 7) of the APS alteration directions (Figure 3G). The data points in the scatter plot of predicted data vs. experimental data were distributed around the diagonal line (Figure 3H). Most (91.4%) of the predicted errors were in a range of ± 0.05 of the normalized APS amplitude (Figure 3I). 

Statistical data showed that during A-HFS with the above three types of IPI sequences repeated in seven rats, the mean RMSEs of the predicted data were smaller than 0.04 of normalized APS amplitude and were significantly smaller than the mean SDs of the experimental APS amplitudes (Figure 3J). Especially for the A-HFS with random IPI, the RMSE = 0.034 ± 0.004 for 133 Hz A-HFS and RMSE = 0.032 ± 0.005 for 150 Hz A-HFS were only half of the corresponding SD = 0.067 ± 0.010 and SD = 0.073 ± 0.012. Similar results were obtained for the pool data of the eight different A-HFS sequences (133 Hz, random IPI) used for establishing the mapping model (see the dashed box in Figure 3J). 

These results indicated that the linear mapping model was able to predict the evoked APS during A-HFS with IPI varying in the range of 5–10 ms. Based on the model, we next developed an algorithm to design pulse sequences for desired neuronal reactions. 

### 3.3. Design Pulse Sequences for Desired APS Distributions

**Figure 4:**
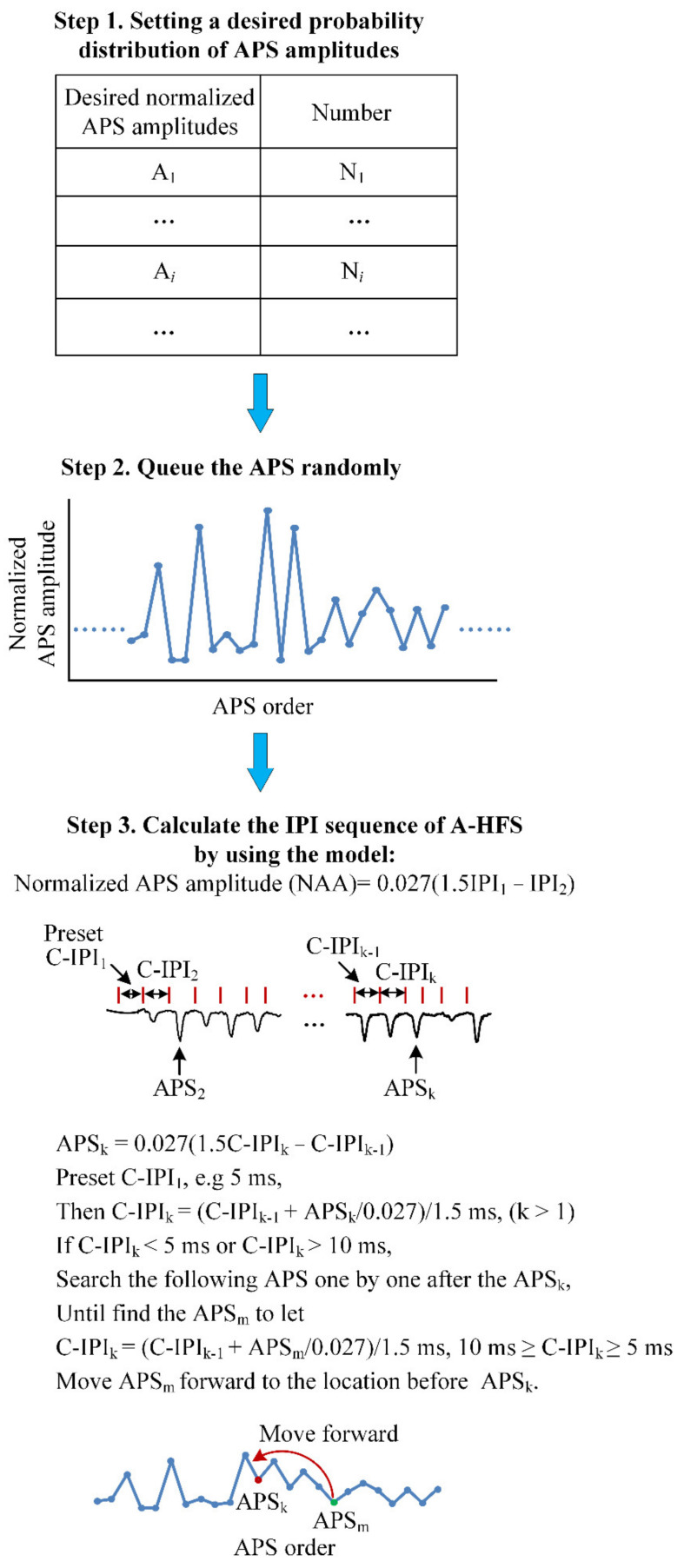
Illustration of the three steps of the algorithm to design a pulse sequence with varying IPI for desired neuronal responses. In step 1, the A*_i_* is the setting amplitude of APS, and N*_i_* is the corresponding number of APS with amplitude A*_i_*.

To obtain a desired distribution of evoked APS, a calculated-IPI (C-IPI) sequence of A-HFS was designed by the following three steps (Figure 4). Step 1: set a desired probability distribution of normalized APS amplitudes with a resolution of 0.000675 determined by the mapping model (Equation (2)) and the sampling rate 20 kHz of experiment recording. Step 2: queue the desired APS amplitudes randomly. Step 3: calculate every C-IPI of the A-HFS based on the mapping model with an initial C-IPI_1_, e.g., 5 ms, and with the APS one by one in its queue. Once a calculated C-IPI is out of the time range 5–10 ms, take the next APS and recalculate the C-IPI, until an APS (termed APS_m_) is found to obtain the C-IPI within 5–10 ms. Move the APS_m_ forward to the location immediately before the current APS (termed APS_k_) and then continue to calculate the next C-IPI by using the APS_k_.

Two different types of desired APS distributions were used to verify the above algorithm.

**Figure 5:**
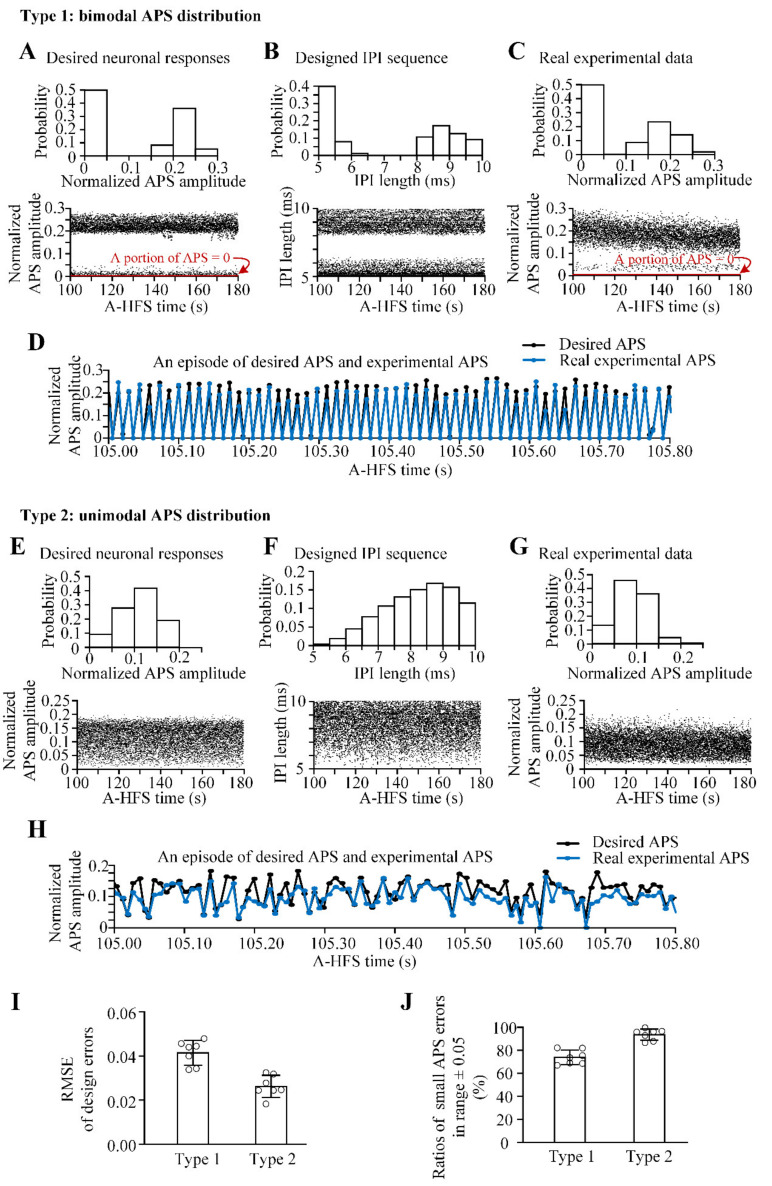
Verification of designed pulse sequences for two different types of desired neuronal responses: Type 1 for a desired APS distribution with bimodal APS amplitudes (**A**–**D**) and Type 2 for a desired APS distribution with unimodal APS amplitudes (**E**–**H**). In (**A**–**C**) and in (**E**–**G**), the top rows are the distributions and the bottom rows are the sequences of the desired APS amplitudes (**A**,**E**), of the designed IPI (**B**,**F**) and of the real experimental APS amplitudes (**C**,**G**). (**D**,**H**) The amplitudes of desired APS and real experimental APS in an episode of A-HFS. (**I)** The design errors (RMSE) of Type 1 and Type 2 (*n* = 7). (**J**) The ratio of small APS errors of Type 1 and Type 2 (*n* = 7), i.e., −0.05 ≤ (the desired APS minus the experimental APS) ≤ 0.05.

Type 1: for a target neuronal reaction with bimodal normalized APS amplitudes distributed in the ranges either smaller (<0.05) or larger (>0.15) and randomly appearing during A-HFS (Figure 5A), the designed IPI sequence distributed mainly in 5–6 and 8–10 ms with a mean pulse frequency of ~140 Hz (Figure 5B). The real distribution of experimental APS amplitudes (Figure 5C) induced by an A-HFS with the designed IPI sequence was similar to the original desired distribution. The experimental APS was able to follow the alterations of desired APS (Figure 5D). 

Type 2: for a target neuronal response with only one peak in the middle of APS distribution (Figure 5E), the distribution of the designed IPI sequence was also unimodal with a mean pulse frequency of 121 Hz (Figure 5F). The real distribution of experimental APS amplitudes (Figure 5G) induced by the designed IPI sequence was similar to the desired unimodal distribution. The alterations of experimental APS were consistent with the desired ones (Figure 5H). 

The above two types of designed IPI sequences were applied in seven experimental rats. The mean RMSE of normalized APS amplitude evoked by the two designed A-HFS sequences were 0.042 ± 0.005 and 0.027 ± 0.005, respectively (Figure 5I). In addition, the ratios of small APS errors in the range of ± 0.05 for Type 1 and Type 2 were 73.9 ± 5.8% and 93.5 ± 4.5%, respectively (Figure 5J), indicating that most of the experimental APS amplitudes were close to the desired APS amplitudes. 

The above results indicated that different types of desired neuronal responses to A-HFS can be approximately obtained through the design of IPI sequences varying in the range of 5–10 ms by using the algorithm.

## 4. Discussion

In the study, a simple linear model was created to describe the relationship between the amplitudes of the evoked APS and the varying IPI of A-HFS. The model was able to predict the neuronal response to each pulse of A-HFS sequences with IPI randomly varying in the range of 5–10 ms, corresponding to 100–200 Hz pulse frequency. Based on the model, an algorithm was created to design pulse sequences for desired neuronal reactions. To our knowledge, the study is the first to propose a quantitative approach to design HFS sequences based on required neuronal reactions with different distributions. The design approach and its implications are analyzed below.

### 4.1. Amplitude Distribution of APS as an Index of the Strength of Stimulation Effects

We applied pulse stimulations at the efferent fibers of principal neurons and utilized the amplitudes of APS waveforms recorded near the cell bodies to evaluate the strength of neuronal responses in the rat hippocampus in vivo (Figure 1A). Neuronal axons are apt to be activated by the narrow pulses commonly used in brain stimulations [27,28]. The APS amplitude can indicate the number of axons synchronously activated by a pulse because the firing of cell bodies is directly induced by the antidromic propagation of axonal activation without involving synaptic transmissions. Additionally, an APS is hardly affected by local inhibitory networks [23], which was confirmed by the similar large APSs induced successionally in the initial period of A-HFS (Figure 1B,F,J). According to the essential cable theory of axons [29], the strengths of activations propagating in both directions are similar. Therefore, the APS amplitude may also indicate the strength of stimulation-induced reactions propagating orthodromically to the terminals of axons, i.e., the pre-synaptic area. Admittedly, through synaptic transmissions, the post-synaptic activity could be different from pre-synaptic activity, which needs further investigations.

A larger APS means activation of a larger population of neurons firing synchronously, thereby representing a stronger neuronal response that could propagate and affect a larger population of neurons in the downstream areas. For a train of APS, even with a similar accumulation amplitude of APS, varied APSs with a few and relatively larger amplitudes can generate stronger neuronal activity in the downstream post-synaptic area than the neuronal activity generated by uniform smaller APSs with constant IPI (see Figure 7 in Feng et al., 2019) [13]. For the two types of designed APS distributions (Figure 5), the amount of relatively large APSs included in bimodal APS distribution (Figure 5C) was greater than the unimodal APS distribution (Figure 5G). The HFS sequence corresponding with the bimodal APS distribution would exert a stronger activation in the downstream area with adequate synaptic transmissions. Therefore, the utilization of the distribution of APS amplitudes as an index to design varying IPI sequences is a feasible strategy and provides a novel approach to adjust the strength of stimulation effects without altering the pulse intensity, i.e., without altering the scope of stimulation action.

### 4.2. Mapping Model Correlating the Neuronal Reactions to the Varying IPI of Stimulation Pulses

During the steady-state period of persistent stimulation of A-HFS with varying IPI in 5–10 ms, each pulse was not able to induce an APS as large as the original one induced at the initial period of A-HFS, but to induce APS varying substantially with amplitudes below 35% of the initial amplitude (Figure 1, Figure 2 and Figure 3). Previous studies with constant IPI in the similar range (i.e., 5–10 ms) have also shown a decrease of APS amplitudes in the steady-state period to approximate 6.1–16.2% of the initial value [24], which conformed with the prediction in the present study with gradually varying IPI (Figure 3D). The attenuation of APS amplitudes may be caused by a putative mechanism of intermittent axonal block induced by HFS [30,31,32]. However, pulses of A-HFS with a constant IPI generate small APS with similar amplitudes, whereas pulses with varying IPI generate varied APS in an enlarged amplitude range because of the non-linear dynamics of neuronal excitations [13,26]. The variations of APS provide an opportunity for designing stimulation sequences for various neuronal reactions. To fulfill the design, we created the linear mapping model (Equation (2)) of APS amplitudes by simply utilizing the two preceding IPIs (IPI_1_ and IPI_2_). 

Because of the intrinsic nonlinear mechanisms of neuronal activation as well as other background inputs to neurons in addition to the applied stimulation, the predicted APS amplitudes had some errors. Nevertheless, the mapping model was able to correctly follow most of the real APS amplitudes (Figure 3). 

Other mapping models including more preceding IPIs and/or with more complex mathematical expressions may not significantly improve the prediction accuracy, because they may introduce more potential errors from additional processes such as more coefficients to be estimated. Moreover, the derivation of the design algorithm could be too complex to realize. In addition, the immediate reaction of neurons following a pulse is mainly correlated with the closest IPIs and is weakly correlated with the IPIs in a longer time distance.

Although the mapping model was established by using the experimental data of varying IPI with a uniform distribution, the model was able to predict the experimental data with other distributions of IPI varying in the identical range of 5–10 ms (Figure 3). Especially for the gradually varying IPI with a relatively long cycle time of tens of seconds, the adjacent IPIs were almost the same. This means that the model was suitable for A-HFS even with constant IPI in the range of 5–10 ms. 

Essentially, the aim of the mapping model was to derive the algorithm for designing pulse sequences with varying IPI from a pre-defined APS distribution for desired neuronal responses (Figure 4). Although the mapping model is linear, solving the inverse problem from an APS distribution to an IPI sequence was a challenge because of infinite solutions. In addition, every IPI must be limited in the range of 5–10 ms. The algorithm developed here was simple, yet skillfully obtained one of the solutions meeting the requirements. The algorithm as well as the mapping model was verified by the results that the distribution of experimental APS induced by a designed pulse sequence conformed to the desired distribution (Figure 5). We used two typical types of desired APS distribution (bimodal and unimodal) with each repeated in the experiments of seven rats. More types of APS distributions are needed to further confirm the robustness of this algorithm.

### 4.3. Implications of the Designs for Different Distributions of Evoked APS Amplitudes

In this study, we limited the varying IPI in the small range of 5–10 ms based on the fact that the commonly used pulse frequency range of brain stimulations (e.g., DBS) is within the range of 100–200 Hz [3,5]. Previous studies have shown that HFS of pulse sequences with a frequency in this range may prevent the propagation of pathological neural signals through possible mechanisms of axonal conduction block and synaptic transmission failure [24,31,33], because continuous HFS can cause the accumulation of potassium outside the axons and result in depolarization block of axon membrane [34,35]. Under this situation, the intrinsic neuronal activity from upstream is masked and replaced by new neuronal activity induced by HFS pulses, which further propagates to the downstream areas [19,20].

Pulse sequences with a constant IPI can generate a mild effect, as each of the pulses only recruits a small portion of neurons [32,36]. However, pulse sequences with varying IPI may synchronously activate a relatively larger portion of neurons with certain randomness as represented by random larger APSs [13,26]. The amount of larger APSs relates with the strength of HFS activation to neurons, acting as a “dose” of stimulations [9]. We showed here that the distribution of APS amplitudes may be designed according to demands by varying IPI in a fixed range but with a variable distribution and a variable order of arrangement (Figure 5). Although the maximum amplitude of varied APS during steady-state period was much smaller than the initial APS amplitudes at the onset of stimulation (Figure 1, Figure 2 and Figure 3), previous studies have shown that the attenuated activation is strong enough to activate the downstream neurons intensely [13]. Additionally, the attenuation may avoid generations of epileptiform activity caused by excessive activation [37]. Presumably, with properly designed sequences of varying IPI, various levels of activations may be obtained below the strength threshold inducing epilepsy. 

In addition, randomness is an important feature in our designs of sequences with varying IPI. Synchronous firing of a larger population of neurons may be otherwise induced by pulses with a regular longer IPI (i.e., a lower pulse frequency) without the need to design specific distribution. However, the stimulation with a lower frequency may fail to mask the intrinsic pathological activity, and would also induce rhythmic activity of neurons, which may cause or aggravate pathological symptoms such as epilepsy and Parkinson’s disease [38,39]. Instead, a high-frequency stimulation with randomness can not only mask the intrinsic neuronal activity, but also modulate neuronal activity to achieve various responses without inducing unwanted rhythmic activity.

The IPI range could be enlarged to include IPI shorter than 5 ms or longer than 10 ms. A larger IPI range may provide more opportunity for generating different APS distributions as long as a new mapping model would be established. However, previous studies have shown that pulses with randomly varying IPI in a range of 5–15 ms may induce the most variable APS events (see Figure 1E in Feng et al., 2019) [13]. A shorter or longer IPI outside this range would result in a smaller or larger APS with more certainty thereby losing the random modulation effects. In addition, long IPIs (i.e., long pauses) may facilitate the conduction of pathological oscillations between brain regions, thereby decreasing the stimulation efficacy [40,41,42]. Nevertheless, previous studies have shown that stimulations with specific low pulse frequencies could also be effective for treating some brain diseases [43,44]. Therefore, it may be worthwhile to further develop the stimulation paradigms of varying IPI with enlarged ranges. 

### 4.4. Limitations of the Study

Although the APS may indicate the original neuronal reactions immediately in the stimulation locations, the final outcomes and clinical efficacy of stimulations relate with the spread of induced neuronal activity and depend on the pathological mechanisms of different brain diseases. Therefore, this study only proposed a potential method to adjust the excitation strength of HFS by varying IPI, which may guide the trials of new stimulation paradigms with varying IPI in animal experiments and in clinical studies. Further studies are needed to reveal the final outcomes of the HFS with different types of varying IPI.

In addition, the study was performed with rats anesthetized by urethane, an anesthetic that has been commonly used in studies of the nervous system in animals [45,46]. The urethane may affect synaptic currents, such as increasing GABAergic currents and decreasing glutamate currents [47], thereby decreasing the firing rate of neurons mildly. Although the antidromic activation used in the present study does not involve synaptic transmissions, the change of neuronal excitability by the anesthetic could affect the evoked APS. Therefore, the parameters of the mapping model as well as the algorithm may need some adjustments for an awake state. Further studies are needed to confirm our results with awake animals. 

## 5. Conclusions

The present study firstly showed that the neuronal response to each pulse of HFS can be designed by changing the inter-pulse-intervals in a small range, which provides a potential approach for programming stimulation patterns to meet the various demands in the application of brain stimulations.

## Data Availability

The data presented in this study are available on request from the corresponding author.
